# Immunogenicity of Multi-Target Chimeric RHDV Virus-like Particles Delivering Foreign B-Cell Epitopes

**DOI:** 10.3390/vaccines10020229

**Published:** 2022-02-02

**Authors:** María Zamora-Ceballos, Noelia Moreno, David Gil-Cantero, José R. Castón, Esther Blanco, Juan Bárcena

**Affiliations:** 1 Instituto Centro de Investigación en Sanidad Animal (CISA-INIA/CSIC), Valdeolmos, 28130 Madrid, Spain; zamora.maria@inia.es (M.Z.-C.); noeliamorm@gmail.com (N.M.); blanco@inia.es (E.B.); 2 Department of Structure of Macromolecules, Centro Nacional de Biotecnología/CSIC, Cantoblanco, 28049 Madrid, Spain; dgil@cnb.csic.es (D.G.-C.); jrcaston@cnb.csic.es (J.R.C.)

**Keywords:** virus-like particles (VLPs), chimeric VLPs, nanoparticles, vaccine platform, multivalent vaccine, RHDV, FCV, CPV, 2L21, B-cell epitope

## Abstract

The rabbit hemorrhagic disease virus (RHDV) vaccine platform is a nanoparticle composed of 180 copies of the viral capsid protein, VP60, self-assembled into virus-like particles (VLPs). RHDV VLPs are able to accept the simultaneous incorporation of target epitopes at different insertion sites. The resulting chimeric RHDV VLPs displaying immunogenic foreign antigens have been shown to induce specific protective immune responses against inserted heterologous T-cytotoxic and B-cell epitopes in the mouse and pig models. In this study, we explored whether RHDV-based engineered VLPs can be developed as efficient multivalent vaccines co-delivering different foreign B-cell antigens. We generated bivalent chimeric RHDV VLPs displaying two model B-cell epitopes at different surface-exposed insertion sites, as well as the corresponding monovalent chimeric VLPs. The immunogenic potential of the bivalent chimeric VLPs versus the monovalent constructs was assessed in the mouse model. We found that the bivalent chimeric VLPs elicited a strong and balanced antibody response towards the two target epitopes tested, although slight reductions were observed in the levels of specific serum antibody titers induced by bivalent chimeric VLPs as compared with the corresponding monovalent constructs. These results suggest that RHDV VLPs could represent a promising platform for the development of efficient multivalent vaccines.

## 1. Introduction

Virus-like particles (VLPs) are nanoparticles built from self-assembled proteins, usually viral capsid subunits, which mimic virion well-defined geometry, while being devoid of genetic material. They are outstanding and safe immunogens combining a highly ordered and particulate nature with a lack of replication ability [1,2,3,4]. Besides their suitability as standalone vaccines for cognate viruses [5,6,7], genetically engineered VLPs are increasingly being explored as vaccine platforms for inducing immune responses against pathogen-derived antigens of choice [8,9,10]. 

Rabbit hemorrhagic disease virus (RHDV) VLPs represent a promising platform for multimeric antigen display [11,12,13,14,15,16] thanks to their intrinsic characteristics: (i) RHDV VLPs are composed of a single capsid subunit, protein VP60 (later termed VP1), easing the production and engineering of chimeric VLPs; (ii) RHDV virions are relatively stable [17]; (iii) RHDV VLPs are highly immunogenic, inducing complete protection of rabbits against RHDV lethal challenge [18]; and (iv) RHDV is restricted to lagomorphs (rabbits and hares), hence no pre-existing immunity is expected in humans or livestock species, avoiding potential detrimental effects of anti-carrier immunity [19,20].

**Figure 1:**
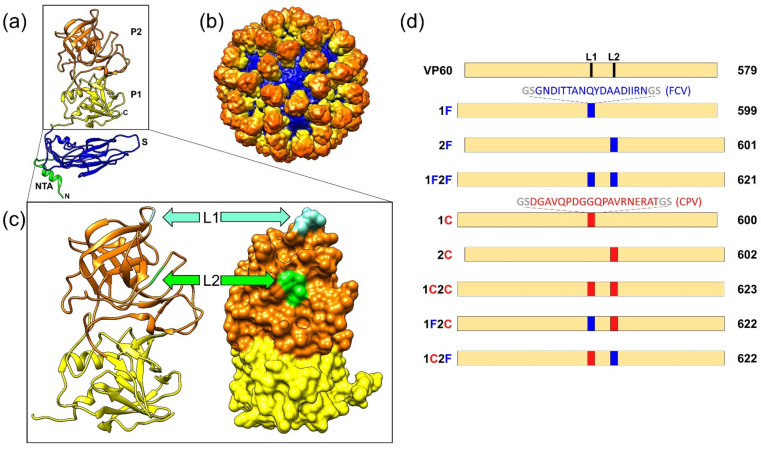
Design of the VP60 insertion mutants generated in this study. (**a**) Ribbon representation of the VP60 protein structure (Protein Data Bank [PDB] accession number 3J1P). The NTA, S domain, and P1 and P2 subdomains are indicated. (**b**) Three-dimensional cryo-EM map of RHDV capsid radially color-coded as in (**a**). (**c**) Ribbon (left) and surface (right) representations of the VP60 P domain. Positions of insertion sites at loops L1 and L2 are indicated. (**d**) Schematic representation of the VP60 chimeric constructs showing names (left) and protein lengths in amino acids (right). The amino acid sequences depicted in blue (FCV B-cell epitope, FCV22) and red (CPV B-cell epitope, 2L21) were incorporated at the indicated positions in each chimeric construct.

The ~40 nm diameter RHDV capsid comprises 90 dimers of the single capsid protein arranged in T = 3 icosahedral symmetry, forming 12 pentamers and 20 hexamers. Each monomer has three domains [21] (Figure 1a), an N-terminal arm (NTA) facing the inner core of the viral capsid, the shell (S) forming a continuous layer protecting the viral genome, and a protruding domain (P) at the outermost surface (Figure 1b) involved in virus—host receptor interactions and antigenic diversity [22]. The P domain is further subdivided into subdomains P1 and P2. The distal P2 subdomain has a β-barrel core formed by β-sheets connected by seven loops (L1–L7) [21] (Figure 1c).

We have previously shown that RHDV VLPs are useful as diagnostic reagents or vaccine candidates for the control of RHDV in rabbits [23,24]. Furthermore, our detailed structural analysis of the RHDV capsid protein enabled the identification of several insertion sites within the primary sequence of the VP60 protein, where foreign immunogenic epitopes can be accommodated without disrupting VLP formation [25,26]. The resulting chimeric VLPs displaying target epitopes have been shown to be excellent immunogens, inducing cellular immune responses against inserted heterologous cytotoxic CD8+ T-cell epitopes [11], as well as potent protective humoral responses against foreign B-cell epitopes in the mouse and pig models [12,13]. RHDV VLPs have proven to be very tolerant, accepting simultaneous insertion of target epitopes at different insertion sites, as well as incorporation of tandem copies of a foreign epitope (up to three, spanning 62 amino acids) at a surface-exposed loop [12].

Building on the RHDV VLP platform proof of concept, this study further explores a relevant issue: whether RHDV-based engineered VLPs can be developed as efficient multivalent vaccines co-delivering different foreign B-cell antigens. Our aim was to evaluate if chimeric RHDV VLPs harbouring two different B-cell epitopes would be able to induce strong humoral responses against both of them, or if immune interference between the target epitopes incorporated could exert a detrimental effect in the foreign antigen-specific immune responses elicited by such bivalent vaccines. We used two model epitopes: a newly described neutralizing B-cell epitope derived from the feline calicivirus (FCV) capsid protein (epitope FCV22) [12,27] and a well characterized B-cell epitope derived from the VP2 capsid protein of canine parvovirus (CPV, epitope 2L21) [28,29]. This epitope is located at the N-terminal end of VP2 and it is well conserved across different related parvoviruses such as feline panleukopenia virus (FPV), mink enteritis virus (MEV), raccoon parvovirus (RPV), and minute virus of mice (MVM) [29,30]. In this study, we designed and constructed bivalent chimeric RHDV VLPs displaying the two model B-cell epitopes at different surface-exposed insertion sites, as well as the corresponding monovalent chimeric VLPs incorporating only one of them. The immunogenic potential of the bivalent chimeric VLPs was analyzed and compared with the monovalent constructs in the mouse model. We found that the bivalent chimeric VLPs elicited a strong and balanced antibody response towards the two target epitopes tested, although slight reductions were observed in the levels of specific serum antibody titers induced by bivalent chimeric VLPs versus the corresponding monovalent constructs. These results suggest the potential suitability of the RHDV VLP platform for the development of efficient multivalent vaccines.

## 2. Materials and Methods

### 2.1. Peptides, Virus, Cells, and Mice

The B-cell epitopes: FCV22 (GSGNDITTANQYDAADIIRN), derived from FCV capsid protein [27], and 2L21 (SDGAVQPDGGQPAVRNERATGS), derived from CPV VP2 protein [29], were prepared as synthetic peptides by solid phase synthesis and HPLC purification (D. Andreu, Pompeu Fabra University, Barcelona, Spain), and were used in ELISA assays to detect specific antibody titers.

Recombinant baculoviruses expressing RHDV VLPs, generated from derivatives of *Autographa californica* nuclear polyhedrosis virus (AcMNPV), were propagated in *Trichoplusia ni* High five cells (H5), grown in monolayer cultures at 28 °C in TNM-FH medium (Sigma-Aldrich, St. Louis, MO, USA), supplemented with 5% fetal calf serum (Gibco, Life Technologies, Thermo Fisher, Waltham, MA, USA).

Feline calicivirus (FCV), Urbana strain, kindly provided by K.Y. Green (NIAID, NIH, Bethesda, MD, USA); canine parvovirus (CPV) CPV2a strain, kindly provided by P. Rueda (Eurofins Ingenasa); and the feline cell line CRFK (Crandell-Reese feline kidney cells; ATCC CCL-94) were used to perform indirect immunofluorescence assays.

C57BL6 female mice (C57BL/6JOlaHsd, Harlan Laboratories in Nederland), 7–8 weeks old, were used to evaluate the immunogenicity of the chimeric VLP constructs.

### 2.2. Generation of Recombinant Baculovirus Transfer Vectors

Plasmid pHAPh306GSopt, containing the gene of VP1 protein (codon-optimized for expression in insect cells), along with the coding sequence of VP2 and the 3′ untranslated region of RHDV (strain AST/89, GenBank accession code Z49271) [24], was used as a template to generate plasmids corresponding to chimeric constructs incorporating amino acid sequences (FCV and/or CPV epitopes) at different locations, by Q5 site-directed mutagenesis (New England Biolabs, Ipswich, MA, USA), according to the manufacturer’s instructions.

DNA sequences corresponding to the FCV-derived epitope (FCV22, GSGNDITTANQYDAADIIRN) and to the CPV-derived epitope (2L21 SDGAVQPDGGQPAVRN-ERATGS), flanked by sequences corresponding to amino acids GS, were incorporated at the insertion sites used in this study: between amino acid positions 306 and 307 (surface-exposed loop L1) and/or between amino acid positions 342 and 343 (surface-exposed loop L2), within the P2 subdomain of RHDV capsid protein (Figure 1c). Plasmids corresponding to eight chimeric constructs were generated (Figure 1d). Two bivalent constructs harboured both epitopes, FCV22 and 2L21, incorporated at loop L1 and loop L2 alternatively (constructs 1F2C and 1C2F), along with three monovalent constructs for each epitope: 1F, 2F, and 1F2F harbouring epitope FCV22, and 1C, 2C, and 1C2C harbouring epitope 2L21. The complete coding sequences of the chimeric VP60 constructs were verified by sequencing.

### 2.3. Construction of Recombinant Baculoviruses

Recombinant baculoviruses were generated using the flashBACULTRA baculoviral genome (Oxford expression technologies, Oxford, UK), following the manufacturer’s instructions.

### 2.4. Expression and Purification of Chimeric RHDV VLPs

Baculovirus infected H5 cell monolayers were harvested after incubation for 4 days at 28 °C, washed three times with 0.2 M phosphate-buffered saline for VLPs (PBS-V: 0.2 M sodium phosphate, 0.1 M NaCl, pH 6.0) and pellets were resuspended in distilled water. After mild sonication and treatment with DNAse I (Roche, Basel, Switzerland), samples were adjusted to 2% Sarkosyl (sodium N-lauroylsarcosine, Sigma), 5 mM EDTA in PBS-V, and incubated overnight at 4 °C. Next, cell lysates were clarified by low-speed centrifugation and the supernatants were centrifuged using a Beckman SW28 rotor at 27,000 rpm for 2 h. Pellets were resuspended in PBS-V, extracted with Vertrel XF (Fluka, Sigma-Aldrich, St. Louis, MO, USA), and centrifuged using a Beckman SW28 rotor at 27,000 rpm for 2 h. The pelleted material was subjected to centrifugation through a 20% (wt/vol) sucrose cushion in PBS-V at 35,000 rpm for 2.5 h using a Beckman SW55 rotor. Finally, the pellets were resuspended in PBS-V containing protease inhibitors (Complete, Roche, Penzberg, Germany) and stored at 4 °C. Protein concentrations of the VLP samples were determined using BCA protein assay kit, Pierce, Thermo Scientific (Waltham, MA, USA).

Recombinant protein expression was analyzed by SDS-polyacrylamide gel electrophoresis (PAGE).

### 2.5. Western Blot Analyses

For Western blot analyses, proteins were transferred from gels onto polyvinylidene difluoride (PVDF) membranes using Trans-Blot^®®^ Turbo™ Transfer System (Bio-Rad Laboratories, Hercules, CA, USA). Membranes were saturated (overnight, 4 °C) with PBS, 5% (wt/vol) skim milk, and 0.5% (vol/vol) Tween 20 and incubated (1 h, 37 °C) with either a rabbit hyperimmune serum against RHDV to detect VP60 protein, 51D10 monoclonal antibody recognizing FCV22 epitope [27], or 3C9 monoclonal antibody against 2L21 epitope [28]. After several washes with PBS–0.05% Tween 20, membranes were incubated (1 h, 37 °C) with HRP-conjugated goat anti-rabbit IgG (Thermo Fisher Scientific, Waltham, MA, USA) or HRP-conjugated goat anti-mouse IgG (Invitrogen, Carlsbad, CA, USA). Membranes were then washed extensively with PBS-0.05% Tween-20 and developed with Pierce ECL Plus Western Blotting (Thermo Fisher Scientific, Waltham, MA, USA). 

### 2.6. Transmission Electron Microscopy

Samples (approximately 5 μL) were applied to glow discharged carbon-coated grids for 2 min and negatively stained with 2% (wt/vol) aqueous uranyl acetate. Images were recorded with a JEOL JEM-1011 electron microscope (JEOL Ltd., Tokyo, Japan) operated at 100 kV, with a 4K × 2.7K ES1000W Erlangshen CCD camera (Gatan, Warrendale, PA, USA) at a nominal magnification of 24,000×.

### 2.7. Mice Immunization

Groups of eight inbred C57BL6 mice (C57BL/6JOlaHsd, Harlan Laboratories in Nederland), female, 7–8 weeks old, were immunized by subcutaneous route at days 0 and 22, with 100 µg of the corresponding chimeric VLP constructs emulsified in Montanide ISA 50V2 (Seppic, Paris, France) in a 1:1 (vol/vol) ratio and sacrificed at day 40 post-immunization. Negative control groups of five mice were inoculated with PBS or the native RHDV VLPs, emulsified with the same adjuvant. Blood samples were collected at day 0 (before priming) and at day 40.

### 2.8. Detection of Specific Antibodies by ELISA

Antibodies against RHDV VP60 capsid protein were determined by ELISA. Here, 300 ng/well of RHDV VLPs diluted in 0.05 M carbonate-bicarbonate buffer (pH 9.6) was incubated at 4 °C overnight in Maxisorp Nunc 96-well plates (Thermo Fisher Scientific, Waltham, MA, USA). After three washes with PBS-0.1%Tween-20, wells were saturated with PBS, 5% (wt/vol) skim milk, for 1 h at 37 °C. Subsequently, sera samples were serially diluted three-fold in PBS and 3% (wt/vol) skim milk and incubated for 1 h at 37 °C. After three washes, plates were incubated with HRP-conjugated goat anti-mouse IgG (Invitrogen, Waltham, MA, USA) at a 1:2000 dilution for 1 h at 37 °C. Finally, plates were extensively washed and colour reaction was developed with TMB (Invitrogen, Waltham, MA, USA). The reaction was stopped by addition of 1.8N H_2_SO_4_ and absorbance at 450 nm was measured on a Fluostar Omega microplate reader. 

Likewise, antibodies against target epitopes FCV22 and 2L21 were determined by ELISA using 96-well High Binding plates (Corning, New York, NY, USA) coated with 5 µg/well of the corresponding synthetic peptide, diluted in 30% 100 mM NH_4_HCO_3_. The procedure was completed as described above for RHDV-specific antibody ELISAs.

Two control samples (serial dilutions) were added to each plate: a monoclonal antibody specific against the target antigen (2E7 for RHDV VP60 protein [24], 51D10 for FCV22 epitope [27], or 3C9 for 2L21 epitope [29]) and a pre-immune serum lacking target antigen-specific antibodies.

End point titers were defined as the highest dilution reciprocal giving an absorbance value greater or equal to 0.2 O.D units above background (the absorbance of wells without antigen, which were consistently in the range of 0.035–0.070, and did not exceed 0.130).

### 2.9. Indirect Immunofluorescence Assays

Indirect immunofluorescence assays (IFAs) were performed to assess the reactivity of the immunized mice sera with the specific antigens (FCV capsid protein and CPV VP2 protein) in the context of viral infection. Feline CRFK cell monolayers, infected either with FCV (Urbana strain) or CPV (CPV2a strain), were fixed with formalin 10% (vol/vol) on coverslips. Once fixed, cells were treated with PBS and 0.2% (vol/vol) triton X-100 for 10 min at room temperature, and then saturated with blocking buffer (PBS, 5% (vol/vol) bovine fetal serum, 1% (wt/vol) bovine serum albumin, and 0.1% (vol/vol) triton-X100) for 30 min. Subsequently, dilutions of sera samples in blocking buffer were incubated with fixed cells for 1 h at room temperature. After washing, Alexa Fluor 488 goat anti-mouse (Life technologies, Thermo Fisher, Waltham, MA, USA) was incubated at 1:500 dilution to detect previous antigen–antibody reactions, for 45 min at room temperature. Fluorescence was preserved using Prolong Gold antifade reagent (Invitrogen, Waltham, MA, USA). The images were obtained with an inverted fluorescence Carl Zeiss Axio Vert A1 microscope.

### 2.10. Statistical Analysis

Data were analyzed using GraphPad Prism Software 6.00 (GraphPad Software, San Diego, CA, USA). Geometric mean titers (GMTs) of IgG in serum were determined for all the groups of mice. One-way analysis of variance (ANOVA), followed by Bonferroni post-hoc comparison tests, were used for comparison of antibody titers among mice groups. All *p*-values were two-sided and *p* < 0.05 was considered statistically significant. In the figures, *p*-value criteria were assigned as follows: ns (not significant) *p* > 0.05, * *p* < 0.05, ** *p* < 0.01, *** *p* < 0.001.

### 2.11. Ethics Statement

The experiments involving mice immunizations were performed at CISA CSIC-INIA animal facilities, in accordance with national and European Union animal experimentation guidelines. The study was previously approved by the Ethical Committee for Animal Experimentation (CEEA 2014/018) and Biosecurity Committee of INIA (CBS 2014/015). Experimental procedures were performed according to protocols approved by the Nacional Committee on Ethics and Welfare (PROEX 228/14).

## 3. Results

### 3.1. Design, Generation, and Characterization of Chimeric RHDV VLPs Displaying FCV and CPV Target Epitopes

We generated a set of recombinant baculoviruses expressing different VP60 insertion mutants incorporating foreign B cell epitopes either at exposed loop L1 and/or exposed loop L2 within P2 subunit of protein VP60 (Figure 1c,d). The target epitopes, FCV22 (22 aa) and 2L21 (23 aa), were flanked by a linker sequence (GS) intended to facilitate accommodation of the foreign sequences inserted. For each epitope, three monovalent constructs were designed, incorporating it alternatively in each one of the two insertion sites tested in this study, or inserting the epitope at both sites (Figure 1d), generating the following constructs: 1F, 2F, and 1F2F (harbouring the FCV epitope) and 1C, 2C, and 1C2C (harbouring the CPV epitope). Additionally, two bivalent constructs were prepared, simultaneously incorporating both epitopes, one at each of the insertion sites, in the two possible configurations: 1F2C and 1C2F.

**Figure 2:**
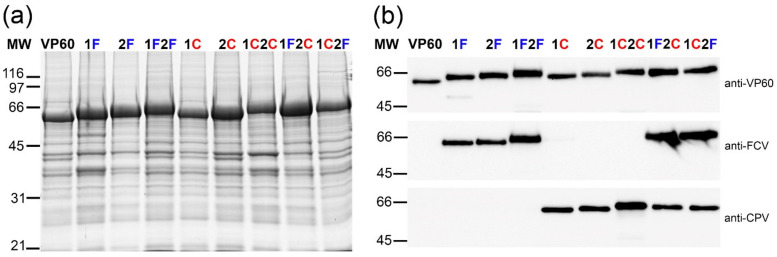
Expression and characterization of RHDV VP60 constructs displaying FCV and CPV target epitopes. (**a**) SDS-PAGE analysis of recombinant baculovirus-infected H5 cell-extracts expressing the indicated constructs. (**b**) Western blots performed using a rabbit hyperimmune serum against RHDV to detect VP60 protein or monoclonal antibodies directed against the FCV and CPV B-cell epitopes, as indicated. The position of molecular weight markers (MW; ×10^3^ Da) is shown on the left.

SDS-PAGE analyses of H5 insect cell cultures infected with the recombinant baculoviruses showed that all the constructs were expressed at grossly similar levels (Figure 2a). Lysates corresponding to cell cultures infected with the baculovirus expressing the VP60 native protein exhibited a major protein band with an apparent molecular weight of ≈60 kDa, whereas the chimeric VP60 constructs displayed slightly slower electrophoretic mobilities, reflecting the presence of peptide sequences corresponding to the inserted target epitopes. 

The infected cell extracts were also subjected to Western blot analysis to verify the presence of the inserted target epitopes (Figure 2b). A rabbit hyperimmune serum against RHDV VLPs reacted with all the VP60-related constructs, while monoclonal antibodies recognized either the FCV or the CPV target epitope, and each reacted with the expected subset of chimeric constructs, according to the presence of the corresponding epitope in each recombinant product.

### 3.2. Electron Microscopy Analysis of the Chimeric RHDV VLPs

**Figure 3:**
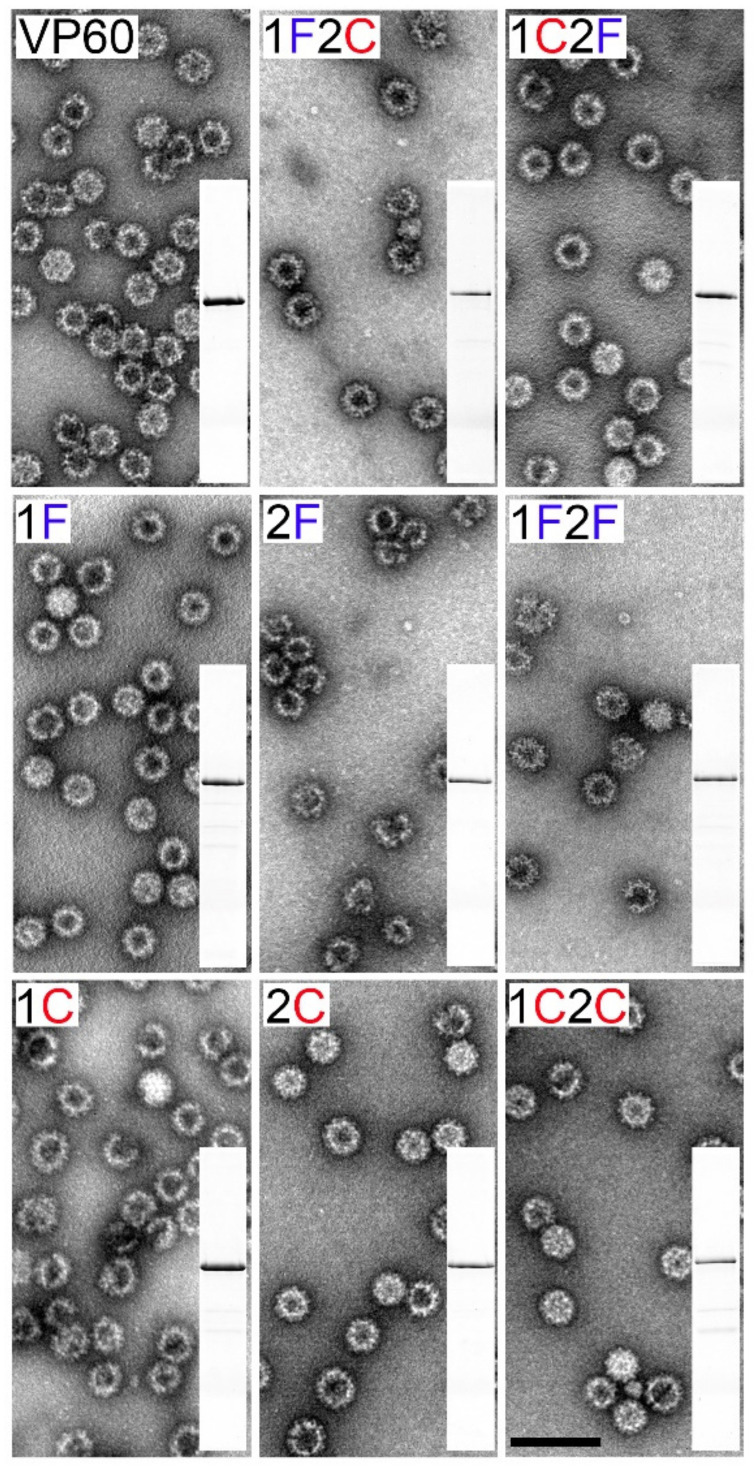
Electron microscopy of negatively stained VP60-related assemblies. Insets show the SDS–PAGE analysis of the purified VP60 chimeric constructs (top, left). Scale bar = 100 nm.

The ability of the novel chimeric constructs to assemble into VLPs was assessed by electron microscopy (Figure 3). Baculovirus infected cell cultures were subjected to VLP-purification procedures and the resulting purified material was analyzed by SDS-PAGE. The samples were found to contain highly purified VP60-related proteins (Figure 3 insets). Negatively stained samples from all chimeric constructs showed VLPs about 40 nm in diameter, with the same morphology as native VP60 VLPs.

### 3.3. Humoral Immune Responses Elicited by Chimeric VLPs Displaying FCV and CPV B-Cell Epitopes in Mice

**Figure 4:**
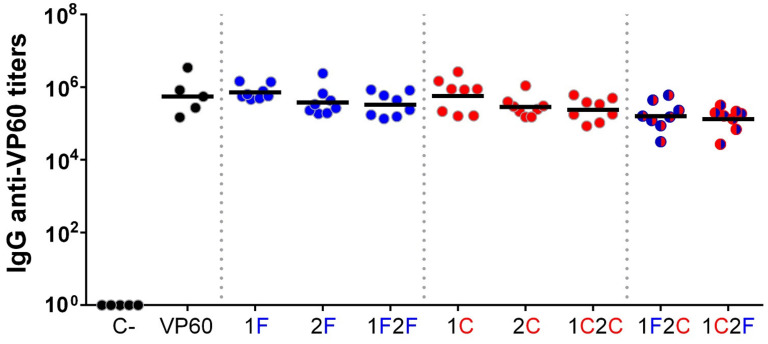
Serum IgG anti-VP60 antibody titers induced by immunization with the chimeric RHDV VLPs. Sera samples from groups of mice inoculated twice with the indicated VLPs were analyzed by ELISA. Anti-VP60 serum IgG antibody titers were determined using RHDV VLPs as antigens. Each symbol represents the value for an individual mouse (*n* = 5 for control groups and *n* = 8 for chimeric VLP groups). GMTs were calculated for all groups (solid lines).

**Figure 5:**
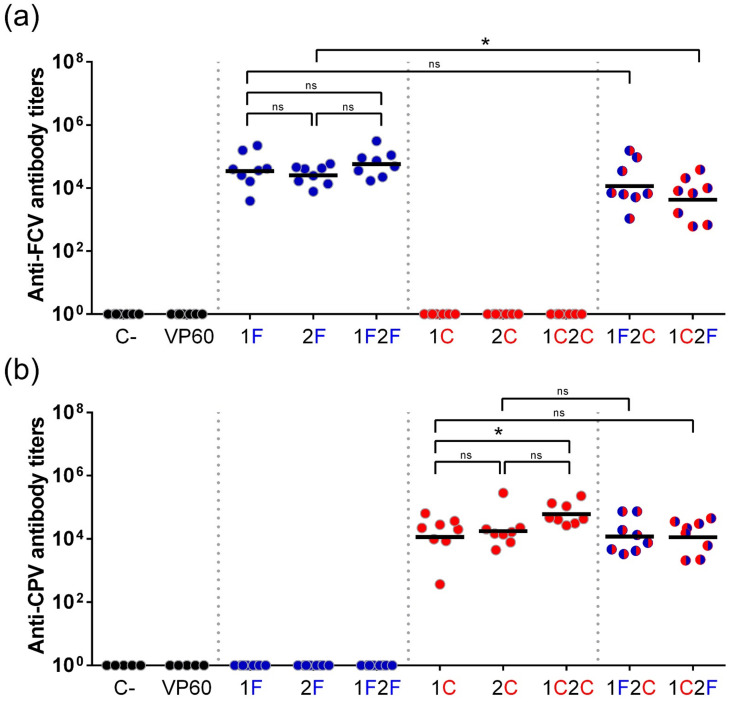
Serum IgG antibody titers against FCV and CPV target epitopes induced by immunization with the chimeric RHDV VLPs. Sera samples from groups of mice inoculated twice with the indicated VLPs were analyzed by ELISA. Anti-FCV (**a**) and anti-CPV (**b**) serum IgG antibody titers were determined using synthetic peptides encompassing the corresponding target epitopes (FCV22 and 2L21, respectively) as antigens. Each symbol represents the value for an individual mouse (*n* = 5 for control groups and *n* = 8 for chimeric VLP groups). GMTs were calculated for all groups (solid lines). * or ns indicates that the differences between the indicated groups are statistically significant (0.01 < *p* < 0.05) or not significant (*p* > 0.05), respectively.

The immunogenicity of chimeric VLPs was evaluated in vivo in the mouse model. Groups of eight C57BL/6 mice were immunized twice subcutaneously with 100 µg of each purified VLP emulsified in Montanide ISA 50V2 adjuvant (groups: 1F, 2F, 1F2F, 1C, 2C, 1C2C, 1F2C, 1C2F). As controls, groups of five mice were immunized with PBS (C-group) or native RHDV VLPs (VP60 group) using the same adjuvant. Sera samples collected at days 0 (preimmune sera) and 40 post-immunization were assayed for antibodies against VP60 protein (RHDV VLPs) (Figure 4) or the FCV and CPV target epitopes (synthetic peptides encompassing FCV22 and 2L21 epitopes) (Figure 5).

Serum IgG antibody titers were measured by ELISA, and geometric mean titers (GMTs) were calculated for all the mice groups. Preimmune and PBS negative control group sera showed no reactivity against the three antigens tested.

All mice immunized with RHDV-derived VLPs elicited high titers of VP60-specific antibodies (GMTs ranging from 1.33 × 10^5^ to 7.22 × 10^5^, Figure 4), as expected, given the reportedly high immunogenicity of RHDV VLPs. The GMTs of the groups corresponding to chimeric VLPs incorporating target epitopes at loop 2 or at both loops were slightly lower than those from groups receiving VLPs displaying epitopes at loop 1.

Regarding the antibody responses elicited against the inserted target epitopes, all chimeric RHDV VLPs displaying the FCV-derived (Figure 5a) or the CPV-derived (Figure 5b) B-cell epitopes, at any of the two insertion sites used, induced specific antibody titers against the foreign epitopes displayed (GMTs ranging from 4.83 × 10^3^ to 6.20 × 10^4^), while sera from mice immunized with PBS (C-group) or native RHDV VLPs (VP60 group) showed no reactivity with the target epitopes.

Regarding the influence of the insertion site (incorporation of target epitope in loop L1 vs. loop L2), no significant differences were observed with both target epitopes tested, as specific antibody titers elicited were similar when comparing groups 1F and 2F (GMTs = 3.69 × 10^4^ and 2.59 × 10^4^, respectively Figure 5a) and groups 1C and 2C (GMTs = 1.31 × 10^4^ and 1.87 × 10^4^, respectively Figure 5b).

Immunization with chimeric VLPs displaying two copies of the same target epitope per VP60 monomer (1F2F and 1C2C) induced higher specific antibody titers than those induced by chimeric VLPs harbouring the target epitope only at one insertion site per monomer, although the titer differences observed were only statistically significant in the case of the chimeric VLPs harbouring the CPV-derived epitope (groups 1C and 1C2C, GMTs = 1.31 × 10^4^ and 6.20 × 10^4^, respectively, Figure 5b). 

Finally, concerning the effect of co-delivery of two different target epitopes, the two bivalent chimeric VLPs (1F2C and 1C2F) were shown to induce high antibody titers against both target epitopes (GMTs = 1.30 × 10^4^ and 4.83 × 10^3^, respectively, against FCV epitope, and GMTs = 1.27 × 10^4^ and 1.21 × 10^4^, respectively, against CPV epitope). When comparing humoral responses elicited by bivalent versus monovalent VLPs, titers induced by bivalent VLPs were shown to be slightly lower, suggesting some degree of immune interference between the target epitopes. However, statistically significant differences were only observed in the case of the responses induced against FCV-derived epitope (groups 2F and 1C2F GMTs = 2.60 × 10^4^ and 4.83 × 10^3^, respectively, Figure 5a), while the GMTs against CPV-derived epitope exhibited by groups of mice immunized with monovalent (1C and 2C) and bivalent (1F2C and 1C2F) VLPs were very similar (GMTs ranging from 1.21 × 10^4^ to 1.87 × 10^4^). These results suggested that the bivalent chimeric RHDV VLPs allowed good immunogenicity for both target epitopes tested.

### 3.4. Evaluation of Sera Reactivity with FCV and CPV Viruses by Immunofluorescence

To assess whether the humoral responses elicited by the chimeric VLPs could react with FCV and CPV viruses in the context of a viral infection, sera samples from the immunized mice were analyzed by indirect immunofluorescence.

**Figure 6:**
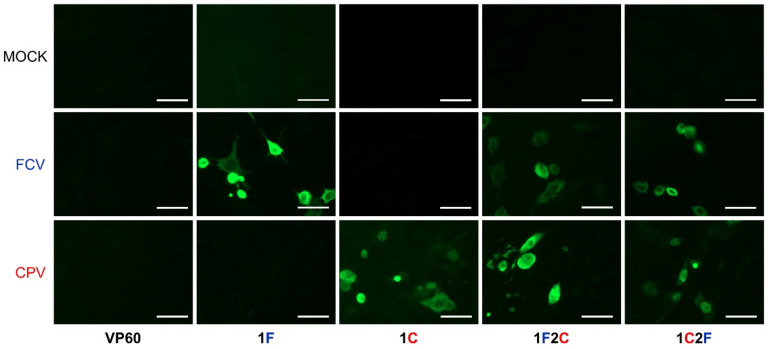
Analysis of sera reactivity with FCV and CPV viruses by immunofluorescence. Indirect immunofluorescence assays were performed using non-infected (mock), FCV-infected (FCV), or CPV-infected (CPV) monolayers of CRFK cells with sera samples of the indicated mice groups. Scale bars = 50 µm.

As shown in Figure 6, the sera tested did not react with uninfected feline CRFK cell monolayers. Sera from mice immunized with native RHDV VLPs showed no reactivity with FCV-infected or CPV-infected CRFK cells. However, sera from groups of mice immunized with monovalent chimeric VLPs (1F and 1C) strongly reacted with the cells infected with the virus matching the corresponding target epitope, while both bivalent chimeric VLPs exhibited potent reactivity with both FCV- and CPV-infected cells. 

## 4. Discussion

VLPs hold promise as efficient tools for a wide array of potential biomedical applications, such as vaccine development, cell targeting, drug delivery, or imaging [31,32,33,34,35,36]. VLP-based vaccines combine key immunogenic properties of viruses with an inherent safety profile [37,38,39].

Several VLP-based platforms have been proposed as scaffolds for multi-target antigen presentation [40,41,42,43,44,45,46,47]. Potential concerns faced by multivalent vaccines are issues related to immune interference and epitope dominance, as numerous reports have shown that co-immunization with multiple antigens can induce immune interference, affecting both antigen specific B cell responses and T cell responses [48,49,50,51,52]. Indeed, in certain cases, multivalent VLPs have failed to induce a strong immune response against an incorporated epitope, in contrast to cognate monovalent VLPs, reflecting problems associated with epitope dominance or inadequate antigen presentation [41,43,45,47]. With this in mind, our aim in this study was to explore the feasibility of developing RHDV-based engineered VLPs as efficient multivalent vaccines co-delivering different foreign B-cell antigens.

Two model epitopes were chosen to perform this task, FCV-derived FCV22 and CPV-derived 2L21, both of which had been previously shown to induce potent humoral immune responses when incorporated to monovalent VLP or virus nanoparticle scaffolds [12,30,53]. The B-cell epitopes were incorporated into RHDV VLPs at two different surface-exposed insertion sites, generating a set of bivalent and monovalent chimeric VLPs, which were subjected to immunogenic evaluation in the mouse model. The eight chimeric constructs generated, displaying one or both target epitopes at one or two different insertion sites within the P2 subdomain of RHDV capsid protein, were efficiently expressed and readily autoassembled into VLPs (Figure 2 and Figure 3). This result further confirmed the reported high versatility of RHDV VLPs as an efficient VLP platform for foreign antigen presentation

Currently, a wide array of chimeric VLPs have been successfully generated using the RHDV platform as a scaffold, incorporating a diversity of B and T cell epitopes from different origins (i.e., FCV, CPV, influenza virus, foot and mouth disease virus, ovalbumin, or cancer-related epitopes), in up to four different insertion sites, or up to three tandem copies at one insertion site (spanning up to 62 amino acids), without disrupting the self-assemble ability of RHDV capsid protein [11,12,13,15,25,54].

Immunization of mice with the chimeric RHDV VLPs elicited high titers of antibodies against the VLP carrier (Figure 4), in agreement with previous results using this VLP plat-form [12,13], and similarly as it happens with other VLP scaffolds [20,55,56,57]. However, this did not preclude the induction of high antibody titers specific against the inserted target epitopes (Figure 5). Further studies are required to evaluate whether previous immunity against carrier VLPs might hamper subsequent uses of this VLP platform. Notably, as RHDV is a lagomorph restricted virus, no pre-existing carrier immunity is expected in humans or relevant livestock species, thus eluding the problem at least for the first time using RHDV VLP-based vaccines.

We tested two insertion sites, incorporating the same target epitopes at each of them (monovalent constructs 1F, 2F, 1C, and 2C). The results obtained indicated both target epitopes induced similar specific antibody titers, regardless of the localization, suggesting the two insertion sites used in this study were similarly efficient for antigen display (Figure 5).

Chimeric VLPs displaying two copies of a target epitope per RHDV capsid monomer (1F2F and 1C2C) induced higher specific antibody titers than those induced by constructs displaying only one copy of the same epitope per monomer (Figure 5), although the titer differences observed were only statistically significant in the case of the chimeric VLPs displaying the CPV-derived epitope. These results were in agreement with previous reports with other chimeric VLPs [20,55].

Finally, concerning the effect of co-delivery of the two different target epitopes, we generated two bivalent chimeric VLPs incorporating both epitopes at the reversed positions (constructs 1F2C and 1C2F) in order to assess whether the inherent immunogenicity of the two epitopes used, or their localization on the VLP capsid surface, was responsible for possible differences detected in the induction of specific antibody titers. This analysis could provide relevant information about the optimal spatial arrangement for the selected target epitopes within the VLP scaffold. As shown in Figure 5, the two bivalent chimeric VLPs were shown to induce high antibody titers against both target epitopes, albeit slightly lower than the corresponding titers induced by the cognate monovalent chimeric VLPs, reflecting some degree of immune interference between the target epitopes. Considering the four possible pairwise comparisons between monovalent and bivalent constructs (1F vs. 1F2C; 2F vs. 1C2F; 1C vs. 1C2F; and 2C vs. 1F2C), only in one case (2F vs. 1C2F, Figure 5a) was a statistically significant difference (*p* < 0.05) observed, indicating that immunity against FCV target epitope incorporated at loop L2 was negatively affected by the presence of CPV epitope at loop L1. However, the detrimental effect was not observed when the target epitopes were allocated at the reversed positions (1F vs. 1F2C). In the case of the CPV epitope, no significant differences were observed irrespective of the insertion site used. The results suggest that different target epitopes may exhibit distinct insertion site preferences, and this should be taken into account in order to obtain optimal specific immune responses.

In summary, the results obtained indicated that the bivalent chimeric RHDV VLPs allowed good simultaneous immunogenicity for both target B-cell epitopes tested, in agreement with previous reports with other chimeric VLPs [42,45,46,47]. Interestingly, RHDV VLPs have been recently reported to efficiently co-deliver two T-cell epitopes (cancer antigens), inducing a targeted anti-tumour immune response [14,15], further extending the feasibility of using the RHDV vaccine platform for the co-delivery of multiple antigens. Therefore, RHDV VLPs may represent a promising platform for the development of multivalent vaccines.

## 5. Conclusions

In this study, we evaluated the feasibility of developing RHDV-based engineered VLPs as multivalent vaccines co-delivering different foreign B-cell antigens. We compared the immune responses elicited in mice by bivalent chimeric RHDV VLPs simultaneously displaying two model B-cell epitopes derived from FCV and CPV, with that elicited by the corresponding monovalent chimeric VLPs. Here, we show that bivalent chimeric RHDV VLPs elicited a strong and balanced antibody response towards the two co-delivered B-cell epitopes, despite slight reductions observed in the levels of specific antibody titers induced as compared with cognate monovalent constructs.

We believe these results further expand the capabilities of an already suitable choice of delivery system, representing an attractive platform for new vaccine development, both in human and the veterinary field.

## Data Availability

All the data that support the findings of this study are included within the article.
